# Effects of non-invasive brain stimulation on walking and balance ability in Parkinson’s patients: A systematic review and meta-analysis

**DOI:** 10.3389/fnagi.2022.1065126

**Published:** 2023-01-10

**Authors:** Xinxin Zhang, Feiyue Jing, Yu’ang Liu, Jinyong Tang, Xianfeng Hua, Jialin Zhu, Haowen Tuo, Qihan Lin, Pincao Gao, Weiguo Liu

**Affiliations:** College of Physical Education and Health, Guangxi Normal University, Guilin, China

**Keywords:** non-invasive brain stimulation, repetitive transcranial magnetic stimulation, transcranial direct current stimulation, Parkinson’s disease, meta-analysis

## Abstract

**Objective:**

To investigate and contrast the effects of non-invasive brain stimulation (NIBS), including repeated transcranial magnetic stimulation (rTMS) and transcranial direct current stimulation (tDCS), on walking and balance ability in patients with Parkinson’s disease (PD).

**Methods:**

The PubMed, Embase, Medline, Cochrane, CNKI, and Chinese WanFang databases were searched up to June 2022. Quality assessment was performed using the Cochrane Collaboration’s risk-of-bias guidelines, and the standardized mean differences (SMD) or mean differences (MD) for each outcome were calculated.

**Results:**

Among 32 eligible studies, including 1,586 participants were analyzed in this meta-analysis. The results of the meta-analysis showed that NIBS was effective in improving UPDRS-III scores (MD = −2.07; 95% CI, −2.62 to −1.53; *P* < 0.00001; *I*^2^ = 6%) and variables associated with the ability of walk such as step width (SMD = 0.35; 95% CI, 0.16–0.55; *P* = 0.0005; *I*^2^ = 38%), cadence (SMD = 0.3; 95% CI, 0.05 to 0.55; *P* = 0.02; *I*^2^ = 25%), and 6MWT (MD = 62.86; 95% CI, 39.43–86.29; *P* < 0.00001; *I*^2^ = 0%). In subgroup analyses across intervention types, UPDRS-III scores (rTMS: MD = −2.54; 95% CI, −3.16 to −1.92; *P* < 0.00001; *I*^2^ = 0%; tDCS: MD = −1.20; 95% CI, −1.99 to −0.40; *P* = 0.003; *I*^2^ = 0%) and TUGT time (rTMS: MD = −4.11; 95% CI, −4.74 to −3.47; *P* < 0.00001; *I*^2^ = 0%; tDCS: MD = −0.84; 95% CI, −1.48 to −0.21; *P* = 0.009; *I*^2^ = 0%) significantly improved. Moreover, our results also showed that compared to tDCS, rTMS was more significant in improving UPDRS-III scores and TUGT time (*p* < 0.05).

**Conclusion:**

NIBS benefits some walking ability variables but not balance ability in 36 patients with PD. The rTMS significantly improved UPDRS-III scores and TUGT time compared to tDCS. Further studies are needed to determine the optimal protocol and to illuminate effects based on the ideal target brain regions, stimulation intensity, timing, and type of intervention.

**Systematic review registration:**

http://www.crd.york.ac.uk/PROSPERO/, identifier CRD42022350782.

## Introduction

Parkinson’s disease (PD) is the second most common neurological degenerative disease and the most common movement disorder, caused by the death of dopamine-producing cells in the substantia nigra (Nascimento et al., 2021). The primary symptoms of PD manifest as movement-related features, including bradykinesia, rigidity, postural instability, and gait disturbances (Obeso et al., 2017). In the last 30 years, the number of people suffering from PD has more than doubled to over 6 million worldwide (GBD 2016 Parkinson’s Disease Collaborators, 2018). The progression associated with medical therapy has led to improvements in motor symptoms such as bradykinesia and rigidity (Morgan et al., 2014). Nonetheless, as the disease develops over time, people with PD face impairments in balance and walking, which are associated with an increased risk of falls, social isolation, and a poorer quality of life (Tomlinson et al., 2013; Bayle et al., 2016). Rehabilitation attempts to preserve and enhance ambulation in the community by improving walking parameters such as gait speed and cadence (Mehrholz et al., 2015).

In current treatment methods, drug administration is the most common choice (Rascol et al., 2002; Connolly and Lang, 2014). However, even with good medical management, patients still possess deterioration in physical function, activities of daily living, and participation (Nijkrake et al., 2007), which can bring about reduced mobility (LaHue et al., 2016) and social isolation (Schrag et al., 2000a), leading to a reduced quality of life (Schrag et al., 2000a). In addition, although surgical techniques, including Deep Brain Stimulation (DBS), can significantly improve the primary motor symptoms of PD (Faggiani and Benazzouz, 2017), less than 5% of patients with PD are eligible for surgery (Morgante et al., 2007).

Neurorehabilitation researchers have recently paid increasing attention to the efficacy of non-invasive brain stimulation (NIBS) as an alternative treatment for motor symptoms in PD (Koch, 2013), which mainly includes repetitive transcranial magnetic stimulation (rTMS) and transcranial direct current stimulation (tDCS) methods (Kim et al., 2019). Neuronal plasticity is the primary mechanism of NIBS for motor symptoms (Kim et al., 2019). The rTMS with frequencies of 5 Hz and above may increase the excitability of the motor core tex, while rTMS at frequencies of 1 Hz and below can temporarily reduce cortical excitability (Koch, 2013). The polarity of the tDCS current influences membrane excitability and changes cortical excitability. DBS has been demonstrated to enhance motor impairments and regulate brain activity and motor cortex physiology in patients with PD (Schlenstedt et al., 2017). NIBS could likewise be utilized as an alternative method to reach the cortex, activating the cortico-basal ganglia-thalamocortical circuit, which has been linked to the pathophysiology of PD (Benninger and Hallett, 2015).

There have been previous studies on the impact of NIBS on PD (Helmich et al., 2006; Benninger and Hallett, 2015; Elsner et al., 2016), as well as several systematic reviews and meta-analyses (Chou et al., 2015; Kim et al., 2019; Lee et al., 2019; Nardone et al., 2020; de Oliveira et al., 2021; Nascimento et al., 2021; Pol et al., 2021; Deng et al., 2022; Krogh et al., 2022). These studies assessed the effect of NIBS on motor symptoms (de Oliveira et al., 2021), dyskinesia (de Oliveira et al., 2021), and gait function (Nardone et al., 2020; Pol et al., 2021; Deng et al., 2022) associated with PD. However, most of these studies were based on independent assessments of the rTMS intervention (Chou et al., 2015; Nardone et al., 2020; Deng et al., 2022; Krogh et al., 2022) and the tDCS intervention (Lee et al., 2019; de Oliveira et al., 2021; Nascimento et al., 2021; Pol et al., 2021) and featured fewer participants. Moreover, these studies did not compare the two interventions or examine them combined. In addition, very few balancing analyses exist, and there were also differences in results between studies. Therefore, the purpose of this meta-analysis was to review the existing literature, comprehensively assess the effects of NIBS on walking and balance ability in patients with PD, and perform subgroup analyses and *Z* tests to elucidate the differences across NIBS stimulation protocols.

## Materials and methods

This review was registered (Identifier: CRD42022350782) in the International Prospective Register of Systematic Reviews (PROSPERO) and complied with the Preferred Reporting Items for Systematic Reviews and Meta-Analyses (PRISMA) statement (Moher et al., 2009).

### Study search and selection

We searched for references on PubMed, Embase, Medline, Cochrane Central Register of Controlled Trials, China National Knowledge Infrastructure (CNKI), and Chinese WanFang databases up to June 2022 without any date and language restrictions. Search terms were: (a) Parkinsonism or PD or Idiopathic Parkinson’s Disease, (b) Transcranial Magnetic Stimulation or Transcranial direct current stimulation or Non-invasive brain stimulation, and (c) Lower limb or Walking or Postural balance or Lower extremity. The inclusion criteria of this meta-analysis included: (a) the study was conducted on patients diagnosed with idiopathic PD, (b) reporting quantitative data related to walking ability or balance, (c) interventions were only NIBS, such as rTMS or tDCS, and (d) using either a crossover design or randomized control trial design. Studies excluded patients with Parkinsonism or Parkinson’s plus disorders; the intervention was DBS, the trial was not conducted with a comparison group, or data on baseline score or end-point outcome were not provided sufficiently. Review articles, editorials, and conferences were also excluded.

The EndNote X9 software was used to remove duplicates from the search, and then two reviewers independently read the titles and abstracts of articles to establish eligibility for inclusion. Studies that failed to meet the inclusion criteria were not reviewed further. Those that could not be excluded were retrieved, and the two reviewers (XZ and JT) assessed the whole text. The authors were contacted *via* email when data validation or more information was required. Disagreements or ambiguities were resolved through a third-reviewer (WL) discussion.

### Data extraction and quality assessment

The following data were gathered from included studies: first author, year of publication, patients’ demographics and clinical presentations, and specific details of experimental design, such as types of interventions, stimulation parameters, total duration of treatment, baseline and end-point outcome measurements, and follow-up time of the study subjects.

The quality of the included studies was assessed using the Cochrane Collaboration’s risk-of-bias guidelines (Higgins et al., 2011). These included: (a) random sequence generation, (b) allocation concealment, (c) blinding of participants and personnel, (d) blinding of outcome assessments, (e) incomplete outcome data, (f) selective reporting, and (g) other biases. If the trial is judged to be at low risk of bias for all domains for this result, it was classified as “low risk of bias” (“+”). The trial was categorized as “high risk of bias” (“–”) if it was assessed to raise some concerns in at least one area for this result but not in any other. If the trial is judged to have a high risk of bias in at least one area for this result, or if the trial is considered to have concerns for many domains in a manner that considerably decreases confidence in the result, it was categorized as having an “uncertain risk of bias” (“?”) (Higgins et al., 2019; de Oliveira et al., 2021). Discrepancies in the evaluations of the two evaluators (XH and HT) were resolved through discussion with a third reviewer (PG).

### Types of outcome measures

The primary outcome parameters included the scores of Part III (motor examination) of the Unified Parkinson’s Disease Rating Scale (UPDRSIII) and the Berg Balance Scale (BBS). Furthermore, the secondary outcome parameters comprised walkability parameters, such as Time Up and Go Test (TUGT), 6 min walking distance (6MWD), stride length, cadence, gait speed, and step width.

### Data synthesis and statistical analysis

All outcome parameters of the studies included in this meta-analysis were continuous variables, so mean differences (MD) or standardized mean differences (SMD) were used as effect sizes by us and were used with 95% confidence intervals (CI) to analyze these studies. The SMD was used when the studies assessed the same outcome but measured it in various ways. Otherwise, the MD was used. The meta-analysis produced effect sizes, which are statistically standardized representations of each study’s quantitative findings (Chung et al., 2006). They were calculated based on the mean pre-post change in the treatment group minus the mean pre-post change in the comparison group, divided by the pooled pretest standard deviation (Feingold, 2009). The RevMan5.2 and Stata14.0 software were used for meta-analysis. R software was used to perform *Z* tests to compare differences in the subgroup’s overall effect sizes based on the intervention type. The Chi^2^ test and *I*^2^ statistic were used to examine study heterogeneity. The fixed-effects model was used if the heterogeneity test did not show statistical significance (*I*^2^< 50%; *p* > 0.05). Otherwise, a random-effects model was used. When there was large heterogeneity in the pooled study results, subgroup analysis and sensitivity analysis were performed. A subgroup analysis according to intervention type was performed. A sensitivity analysis was conducted to prove the reliability of our meta-analysis results by removing each study to evaluate the consistency and quality of the results (Gao et al., 2021). When there were more than 10 studies with outcome indicators, funnel plots, and Egger asymmetry tests were used to assess for publication bias. It was hard to find the cause of asymmetry when there were fewer than ten studies (Sterne et al., 2011). Statistically significant differences were set at α = 0.05.

## Results

### Search results

**FIGURE 1:**
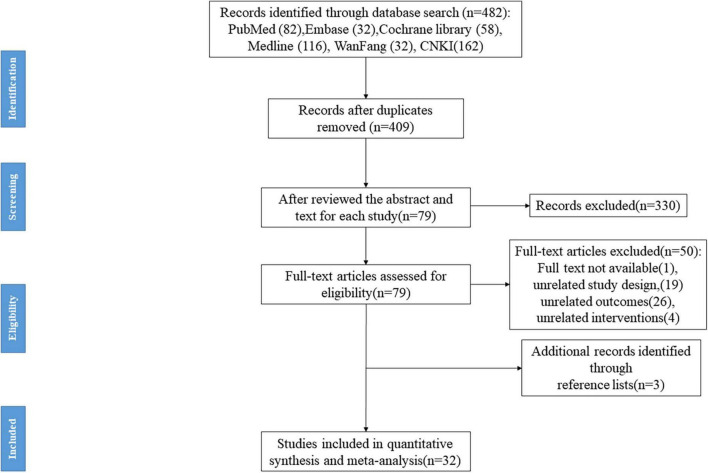
The flow diagram of the selection process.

A total of 482 records were retrieved, and after removing duplicates with EndNote, 409 records were included in the initial screening. Three reviewers reviewed the abstract and text for each study based on our inclusion and exclusion criteria. We excluded 330 studies because of review articles, unavailability of full text, and unrelated study design, interventions, and outcome parameters (e.g., protocol studies). Seventy-nine records were included in the full-text screening. Through screening, we excluded another 50 studies that did not fit the criteria. However, we found three studies in the 79 records list of references that met our inclusion criteria, so we included them. In the end, 32 studies met the inclusion criteria (Benninger et al., 2010, 2011, 2012; Maruo et al., 2013; Yang et al., 2013; Chen et al., 2014; Kaski et al., 2014; Kim et al., 2015; Tang et al., 2015; Chang et al., 2016, 2017; Li, 2016; Schabrun et al., 2016; Swank et al., 2016; Costa-Ribeiro et al., 2017; Yu et al., 2017; Qiao et al., 2018, 2019; Yotnuengnit et al., 2018; Bueno et al., 2019; Wang et al., 2019; Wu et al., 2019; Zhang et al., 2020; Chen and Li, 2021; Guo, 2021; Hu et al., 2021; Huang et al., 2021; Liu and Zhang, 2021; Zhang and Mao, 2021; Li et al., 2022; Ren and He, 2022; Wong et al., 2022), of which 16 studies (Benninger et al., 2010, 2011, 2012; Maruo et al., 2013; Yang et al., 2013; Kaski et al., 2014; Kim et al., 2015; Chang et al., 2016, 2017; Schabrun et al., 2016; Swank et al., 2016; Costa-Ribeiro et al., 2017; Yotnuengnit et al., 2018; Bueno et al., 2019; Wang et al., 2019; Wong et al., 2022) were published in English, and the other 16 studies (Chen et al., 2014; Tang et al., 2015; Li, 2016; Yu et al., 2017; Qiao et al., 2018, 2019; Wu et al., 2019; Zhang et al., 2020; Chen and Li, 2021; Guo, 2021; Hu et al., 2021; Huang et al., 2021; Liu and Zhang, 2021; Zhang and Mao, 2021; Li et al., 2022; Ren and He, 2022) were published in Chinese. The same research center published several studies included in the meta-analysis. In order to avoid the inclusion of duplicate samples, these studies were carefully examined at the inclusion stage (including the age of the patient and intervention protocols). The flowchart is shown in Figure 1.

### Participant characteristics

This meta-analysis included 32 studies, including 1,586 patients with PD (range of mean age = 50.09–71.9 years), and the mean age of Parkinson’s patients in one study (Kaski et al., 2014) was not reported. The mean time duration after PD diagnosis ranged from 1.64 to 10.8 years, and seven studies (Maruo et al., 2013; Kaski et al., 2014; Wang et al., 2019; Hu et al., 2021; Liu and Zhang, 2021; Zhang and Mao, 2021; Wong et al., 2022) were not reported. The Hoehn and Yahr scale ranged from 1 to 5. Twenty-seven of the 32 total studies reported participants’ medication status, with all reported studies indicating that patients with PD were on medication (medication use and dosage were consistent across the intervention and control groups) and the remaining five studies (Kim et al., 2015; Bueno et al., 2019; Wang et al., 2019; Huang et al., 2021; Wong et al., 2022) not reporting medication use. Specific details regarding participant characteristics are shown in Table 1.

**TABLE 1:** The detailed characteristics of each included study.

References	Study design	Sample size	Sex (M/F)	Age (Mean ± SD)	PD duration, years (Mean ± SD)	Medication	H and Y stage
Benninger et al. (2011)	Parallel	E: 13	7/6	62.1 ± 6.9	10.8 ± 7.1	ON	2–4
		C: 13	11/2	65.6 ± 9.0	6.5 ± 3.4		
Bueno et al. (2019)	Crossover	Total: 20	12/8	64.45 ± 8.98	7.80 ± 5.32	NR	1.5–3
Chang et al. (2016)	Crossover	Total: 8	6/2	71.9 ± 7.8	4.3 ± 1.8	ON	NR
Chen et al. (2014)	Parallel	E-L: 21	10/11	65.81 ± 9.38	5.88 ± 5.29	ON	NR
		E-H: 21	10/11	63.19 ± 8.16	6.60 ± 5.53		
		C: 18	9/9	66.61 ± 8.00	6.22 ± 3.96		
Chen and Li (2021)	Parallel	E: 52	30/22	66.96 ± 11.46	3.54 ± 1.21	ON	1–4
		C: 52	29/23	66.32 ± 5.27	3.41 ± 1.13		
Costa-Ribeiro et al. (2017)	Parallel	E: 11	NR	61.1 ± 9.1	6.1 ± 3.8	ON	1–3
		C: 11		62.0 ± 16.7	6.3 ± 3.7		
Benninger et al. (2010)	Parallel	E: 13	9/4	63.66 ± 9.0	10.66 ± 7.1	ON	2–4
		C: 12	7/5	64.26 ± 8.8	9.16 ± 3.3		
Benninger et al. (2012)	Parallel	E: 13	11/2	55.8 ± 9.1	8.6 ± 4.1	ON	2–4
		C: 13	9/4	54.3 ± 12.5	9.3 ± 6.8		
Guo (2021)	Parallel	E: 33	23/10	62.02 ± 6.79	2.61 ± 0.68	ON	1–2.5
		C: 32	24/8	60.71 ± 7.38	2.53 ± 0.70		
Hu et al. (2021)	Parallel	E: 49	28/21	63.68 ± 5.22	NR	ON	1–5
		C: 49	30/19	64.23 ± 4.78			
Huang et al. (2021)	Parallel	E: 30	18/12	60.15 ± 3.15	3.66 ± 1.63	NR	NR
		C: 30	17/13	60.13 ± 3.13	3.59 ± 1.59		
Kaski et al. (2014)	Crossover	Total: 8	NR	NR	NR	ON	NR
Kim et al. (2015)	Crossover	Total: 17	12/5	64.5 ± 8.4	7.8 ± 4.9	NR	2.5–4
Li (2016)	Parallel	E-L: 30	16/14	66.1 ± 7.6	6.1 ± 5.2	ON	NR
		E-H: 30	15/15	65.3 ± 8.1	6.6 ± 5.3		
		C: 30	16/14	66.5 ± 7.5	6.4 ± 4.9		
Li et al. (2022)	Parallel	E: 30	19/11	62.36 ± 9.26	7.82 ± 3.31	ON	1–4
		C: 30	17/13	60.87 ± 8.93	6.79 ± 2.86		
Liu and Zhang (2021)	Parallel	E: 30	18/12	59.7 ± 9.1	NR	ON	NR
		C: 30	16/14	60.2 ± 8.9			
Maruo et al. (2013)	Crossover	Total: 21	10/11	63.0 ± 11.3	NR	ON	2–4
Qiao et al. (2018)	Parallel	E: 15	7/8	62.00 ± 10.26	4.03 ± 4.94	ON	1–2.5
		C: 15	9/6	58.40 ± 9.13	3.51 ± 2.51		
Qiao et al. (2019)	Parallel	E: 25	11/14	63.00 ± 9.20	5.22 ± 4.46	ON	1–3
		C: 24	15/9	62.04 ± 9.69	6.07 ± 5.25		
Ren and He (2022)	Parallel	E: 25	14/11	61.6 ± 1.3	4.2 ± 0.8	ON	1–3
		C: 25	15/10	61.2 ± 1.1	4.1 ± 0.6		
Schabrun et al. (2016)	Parallel	E: 8	8/0	72 ± 4.9	6.9 ± 4.4	ON	2–3
		C: 8	6/2	63 ± 11.0	4.6 ± 3.9		
Swank et al. (2016)	Crossover	Total: 10	8/2	68.7 ± 10.2	7.9 ± 7.1	ON	NR
Tang et al. (2015)	Parallel	E: 25	Total:27/23	Total: 68.5 ± 11.2	6.8 ± 2.5	ON	1–3
		C: 25					
Wang et al. (2019)	Parallel	E: 8	7/1	53.5 ± 13.7	NR	NR	NR
		C: 6	4/2	54.7 ± 12.2			
Chang et al. (2017)	Parallel	E: 16	9/7	63.6 ± 7.5	9.8 ± 4.7	ON	1.5–4
		C: 16	11/5	63.8 ± 8.3	9.1 ± 5.3		
Wong et al. (2022)	Parallel	M1 group: 9	8/1	54.20 ± 4.1	NR	NR	1–3
		DLPFC group: 9	6/3	50.09 ± 2.4			
		Cerebellum group: 9	2/7	61.30 ± 7.9			
		C:9	3/6	58.30 ± 8.0			
Wu et al. (2019)	Parallel	E: 50	30/20	59.6 ± 6.1	5.8 ± 1.6	ON	1–3
		C: 50	31/19	60.2 ± 6.3	6.0 ± 1.7		
Yang et al. (2013)	Parallel	E: 10	5/5	65.20 ± 11.08	6.40 ± 2.76	ON	2–3
		C: 10	7/3	67.00 ± 13.21	6.35 ± 3.58		
Yotnuengnit et al. (2018)	Parallel	tDCS: 18	10/8	64.4 ± 7.8	7.9 ± 3.9	ON	2–3
		tDCS + PT: 17	11/6	68.2 ± 9.8	9.4 ± 5.3		
		Physical therapy:18	12/6	62.7 ± 8.8	6.6 ± 3.6		
Yu et al. (2017)	Parallel	E: 31	NR	60.32 ± 9.63	2.62 ± 0.86	ON	1–2
		C: 30		60.16 ± 10.14	1.64 ± 0.52		
Zhang and Mao (2021)	Parallel	E: 111	62/49	63.8 ± 6.1	NR	ON	1–2.5
		C: 110	57/53	64.3 ± 6.8			
Zhang et al. (2020)	Parallel	E: 37	21/16	59.42 ± 5.02	3.62 ± 0.81	ON	1–3
		C: 37	20/17	59.74 ± 4.84	3.45 ± 0.77		

E, experimental group; C, control group; NR, not reported.

### Intervention protocols

The specific parameters of the rTMS and tDCS interventions are shown in Tables 2, 3. In 18 (Benninger et al., 2011, 2012; Maruo et al., 2013; Yang et al., 2013; Chen et al., 2014; Kim et al., 2015; Tang et al., 2015; Chang et al., 2016; Li, 2016; Yu et al., 2017; Wang et al., 2019; Wu et al., 2019; Zhang et al., 2020; Chen and Li, 2021; Guo, 2021; Huang et al., 2021; Li et al., 2022; Ren and He, 2022) of the 32 studies, rTMS was utilized as an intervention, whereas the remaining 14 (Benninger et al., 2010; Kaski et al., 2014; Schabrun et al., 2016; Swank et al., 2016; Chang et al., 2017; Costa-Ribeiro et al., 2017; Qiao et al., 2018, 2019; Yotnuengnit et al., 2018; Bueno et al., 2019; Hu et al., 2021; Liu and Zhang, 2021; Zhang and Mao, 2021; Wong et al., 2022) used tDCS. Of the 18 rTMS intervention studies, seven studies (38.89%) (Benninger et al., 2011, 2012; Chen et al., 2014; Kim et al., 2015; Chang et al., 2016; Yu et al., 2017; Wang et al., 2019) reported follow-up, 15 (83.3%) (Benninger et al., 2011, 2012; Yang et al., 2013; Chen et al., 2014; Tang et al., 2015; Li, 2016; Yu et al., 2017; Wang et al., 2019; Wu et al., 2019; Zhang et al., 2020; Chen and Li, 2021; Guo, 2021; Huang et al., 2021; Li et al., 2022; Ren and He, 2022) utilized a parallel design, and seven (38.89%) (Yang et al., 2013; Wu et al., 2019; Zhang et al., 2020; Chen and Li, 2021; Guo, 2021; Huang et al., 2021; Li et al., 2022) combined rTMS with other therapies. Three studies (Tang et al., 2015; Chen and Li, 2021; Guo, 2021) applied low-frequency stimulation; 10 (Benninger et al., 2011, 2012; Maruo et al., 2013; Yang et al., 2013; Kim et al., 2015; Chang et al., 2016; Yu et al., 2017; Wang et al., 2019; Zhang et al., 2020; Ren and He, 2022) applied high-frequency stimulation, and five (Chen et al., 2014; Li, 2016; Wu et al., 2019; Huang et al., 2021; Li et al., 2022) used a mix of high-frequency and low-frequency stimulation. rTMS stimulation of brain areas included the prefrontal cortex (PFC) (Huang et al., 2021; Li et al., 2022), the dorsolateral prefrontal lobe cortex (DLPFC) (Li, 2016; Yu et al., 2017; Wu et al., 2019; Zhang et al., 2020; Ren and He, 2022), the frontal right lobe (FRL) (Chen and Li, 2021), the primary motor cortex (M1) (Benninger et al., 2012; Maruo et al., 2013; Chen et al., 2014; Tang et al., 2015; Guo, 2021), and the primary motor cortex of the lower leg (M1-LL) (Yang et al., 2013; Kim et al., 2015; Chang et al., 2016; Wang et al., 2019), with one study (Benninger et al., 2011) combining M1 and DLPFC stimulation protocols. On the other hand, only five (Benninger et al., 2010; Schabrun et al., 2016; Chang et al., 2017; Costa-Ribeiro et al., 2017; Yotnuengnit et al., 2018) of the 16 (31.25%) tDCS intervention studies recorded the follow-up, 11 (68.75%) (Benninger et al., 2010; Schabrun et al., 2016; Chang et al., 2017; Costa-Ribeiro et al., 2017; Qiao et al., 2018, 2019; Yotnuengnit et al., 2018; Hu et al., 2021; Liu and Zhang, 2021; Zhang and Mao, 2021; Wong et al., 2022) adopted a parallel design, and one research (Yotnuengnit et al., 2018) combined tDCS with physical therapy.

**TABLE 2:** The specific parameters of rTMS interventions.

References	Study design	Intervention type	Stimulation parameters					The total duration of treatment	Follow up
			Frequency	Intensity	Coil type	Sites	No. of plus		
Benninger et al. (2011)	Parallel	rTMS	50 Hz	80%AMT	C	M1 + DLPFC	8,000	2 weeks	Yes (1 month)
Chang et al. (2016)	Crossover	rTMS	10 Hz	90%RMT	C	M1-LL	1,000	5 days	Yes (12 days)
Chen et al. (2014)	Parallel	rTMS	1, 5 Hz	100%RMT	F8	M1	1,600	10 days	Yes (1 month)
Chen and Li (2021)	Parallel	rTMS + Levodopa and Benserazide Hydrochlo-ride	1 Hz	60%AMT	F8	FRL	NR	2 months	No
Benninger et al. (2012)	Parallel	rTMS	50 Hz	80%AMT	NR	M1	4,800	2 weeks	Yes (1 month)
Guo (2021)	Parallel	rTMS + Pramipexole	0.5 Hz	90%RMT	C	M1	3,200	4 weeks	No
Huang et al. (2021)	Parallel	rTMS + Rehabilitation Training	1, 5 Hz	80%AMT	F8	PFC	800	1 month	No
Kim et al. (2015)	Crossover	rTMS	10 Hz	90%RMT	C	M1-LL	1,000	5 days	Yes (12 days)
Li (2016)	Parallel	rTMS	0.5, 5 Hz	90∼100%AMT	NR	DLPFC	750	4 weeks	No
Li et al. (2022)	Parallel	rTMS + Pramipexole	1, 5 Hz	80%AMT	NR	PFC	NR	3 months	No
Maruo et al. (2013)	Crossover	rTMS	10 Hz	100%RMT	F8	M1	3,000	3 days	No
Ren and He (2022)	Parallel	rTMS	10 Hz	90%AMT	F8	DLPFC	800	4 weeks	No
Tang et al. (2015)	Parallel	rTMS	1 Hz	120%RMT	F8	M1	250	1 month	No
Wang et al. (2019)	Parallel	rTMS	5 Hz	90%RMT	F8	M1-LL	900	3 weeks	Yes (1 month)
Wu et al. (2019)	Parallel	rTMS + Rehabilitation Training	1, 5 Hz	80%AMT	C	DLPFC	1,600	4 weeks	No
Yang et al. (2013)	Parallel	rTMS + Treadmill training	5 Hz	100%RMT	F8	M1-LL	1,200	4 weeks	No
Yu et al. (2017)	Parallel	rTMS	5 Hz	100%AMT	F8	DLPFC	800	10 days	Yes (1 month)
Zhang et al. (2020)	Parallel	rTMS + Rehabilitation Training	10 Hz	NR	F8	DLPFC	800	3 months	No

rTMS, repetitive transcranial magnetic stimulation; M1, primary motor cortex; DLPFC, dorsolateral prefrontal cortex; AMT, active motor threshold; C, circular; RMT, resting motor threshold; F8, figure of 8; M1-LL, primary motor cortex of the lower leg; PFC, prefrontal cortex; FRL, frontal right lobe; NR, not reported.

**TABLE 3:** The specific parameters of tDCS interventions.

References	Study design	Intervention type	Stimulation parameters					The total duration of treatment	Follow up
			Session	Anodal electrode site	Intensity	Duration	Areas		
Bueno et al. (2019)	Crossover	tDCS	2	DLPFC	2 mA	20 min	35 cm^2^	NR	No
Costa-Ribeiro et al. (2017)	Parallel	tDCS	NR	MC	2 mA	13 min	35 cm^2^	4 weeks	Yes (1 month)
Benninger et al. (2010)	Parallel	tDCS	8	PMC, MC	2 mA	20 min	97.5 cm^2^	2.5 weeks	Yes (1, 3 months)
Hu et al. (2021)	Parallel	tDCS	1	DLPFC	2 mA	25 min	35 cm^2^	NR	No
Kaski et al. (2014)	Crossover	tDCS	NR	M1	2 mA	15 min	40 cm^2^	NR	Nr
Liu and Zhang (2021)	Parallel	tDCS	1	Bi	1.4 mA	20 min	24 cm^2^	14 days	No
Qiao et al. (2018)	Parallel	tDCS	1	DLPFC	2 mA	20 min	NR	5 days	No
Qiao et al. (2019)	Parallel	tDCS	1	DLPFC	2 mA	20 min	35 cm^2^	5 days	No
Schabrun et al. (2016)	Parallel	tDCS	9	M1	2 mA	20 min	35 cm^2^	3 weeks	Yes (12 weeks)
Swank et al. (2016)	Crossover	tDCS	2	DLPFC	2 mA	20 min	NR	7 ± 2 days	No
Chang et al. (2017)	Parallel	tDCS	5	DLPFC	1 mA	20 min	NR	5 days	Yes (12 weeks)
Wong et al. (2022)	Parallel	tDCS	NR	M1, DLPFC, Cerebellum	2 mA	20 min	35 cm^2^	NR	No
Yotnuengnit et al. (2018)	Parallel	tDCS + Physical Therapy	6	MC	2 mA	30 min	35 cm^2^	2 weeks	Yes (2, 4, 8 weeks)
Zhang and Mao (2021)	Parallel	tDCS	1	DLPFC	2 mA	20 min	NR	20 days	No

tDCS, transcranial direct current stimulation; Bi, bilateral brain region; DLPFC, dorsolateral prefrontal cortex; M1, primary motor cortex; PMC, premotor cortex; MC, motor cortex; NR, not reporting.

Targeted brain regions of active tDCS included the premotor cortex (PMC) (Benninger et al., 2010), the motor cortex (MC) (Benninger et al., 2010; Costa-Ribeiro et al., 2017; Yotnuengnit et al., 2018), the DLPFC (Swank et al., 2016; Chang et al., 2017; Qiao et al., 2018, 2019; Bueno et al., 2019; Hu et al., 2021; Zhang and Mao, 2021; Wong et al., 2022), the bilateral brain region (Bi) (Liu and Zhang, 2021), the M1 (Kaski et al., 2014; Schabrun et al., 2016; Wong et al., 2022), and the cerebellum (Wong et al., 2022). Lastly, three studies (Kaski et al., 2014; Costa-Ribeiro et al., 2017; Wong et al., 2022) did not describe sessions of tDCS procedures, while four studies did not report stimulation parameters (Swank et al., 2016; Chang et al., 2017; Qiao et al., 2018; Zhang and Mao, 2021) and total treatment duration (Kaski et al., 2014; Bueno et al., 2019; Hu et al., 2021; Wong et al., 2022).

### Methodological quality assessment

**FIGURE 2:**
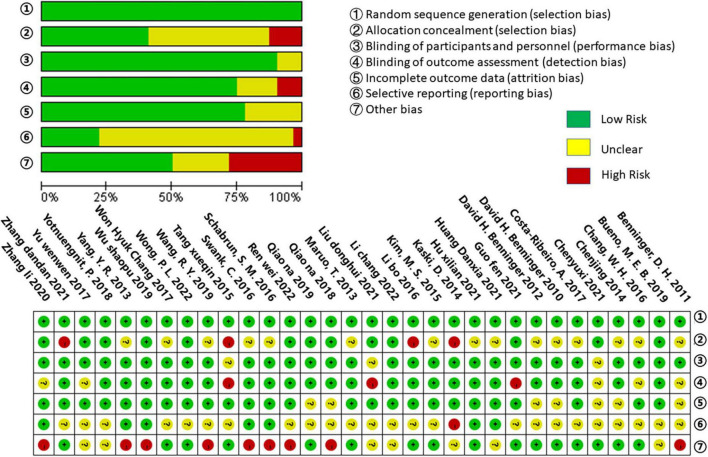
Risk of bias summary.

The methodological quality of all included studies was evaluated using the Cochrane Collaboration’s techniques for assessing bias risk. All of the included trials described randomized allocation and were low-risk in the fields of randomized allocation. In allocation concealment, 15 studies (Benninger et al., 2010, 2011, 2012; Maruo et al., 2013; Yang et al., 2013; Chen et al., 2014; Kim et al., 2015; Chang et al., 2016, 2017; Schabrun et al., 2016; Swank et al., 2016; Costa-Ribeiro et al., 2017; Wang et al., 2019; Hu et al., 2021; Huang et al., 2021) were classified as having an unclear risk, while four (Kaski et al., 2014; Tang et al., 2015; Li, 2016; Zhang and Mao, 2021) were classified as having a high risk. In the domain of blinding of participants and personnel, there was a lower risk of bias; just three studies (Tang et al., 2015; Chen and Li, 2021; Liu and Zhang, 2021) were assessed as having an unclear risk, while the rest had a low risk. In their outcome assessment, eight studies (Benninger et al., 2011; Tang et al., 2015; Chang et al., 2016; Costa-Ribeiro et al., 2017; Yu et al., 2017; Zhang et al., 2020; Guo, 2021; Liu and Zhang, 2021) were not blind, and three (Tang et al., 2015; Guo, 2021; Liu and Zhang, 2021) were classified as high risk. Seven studies (Benninger et al., 2010, 2011, 2012; Chen et al., 2014; Qiao et al., 2018, 2019; Chen and Li, 2021) reported methods with an unclear risk of incomplete outcome data. Concerning selective outcome reporting bias, 24 studies (Benninger et al., 2010, 2011, 2012; Maruo et al., 2013; Chen et al., 2014; Kim et al., 2015; Tang et al., 2015; Chang et al., 2016, 2017; Li, 2016; Costa-Ribeiro et al., 2017; Yu et al., 2017; Qiao et al., 2018, 2019; Yotnuengnit et al., 2018; Bueno et al., 2019; Wang et al., 2019; Chen and Li, 2021; Guo, 2021; Liu and Zhang, 2021; Zhang and Mao, 2021; Li et al., 2022; Ren and He, 2022; Wong et al., 2022) were deemed to be at unclear risk, while one study (Kaski et al., 2014) was deemed to be at high risk. Nine studies (Benninger et al., 2011; Yang et al., 2013; Schabrun et al., 2016; Swank et al., 2016; Qiao et al., 2018; Wang et al., 2019; Wu et al., 2019; Zhang et al., 2020; Ren and He, 2022) were assessed as having a high risk of other bias, and seven (Kaski et al., 2014; Yu et al., 2017; Yotnuengnit et al., 2018; Bueno et al., 2019; Huang et al., 2021; Liu and Zhang, 2021; Li et al., 2022) were evaluated as having an unclear risk. These results are summarized in Figure 2.

### Meta-analytic results

#### Effects of NIBS on UPDRS-III scores

**FIGURE 3:**
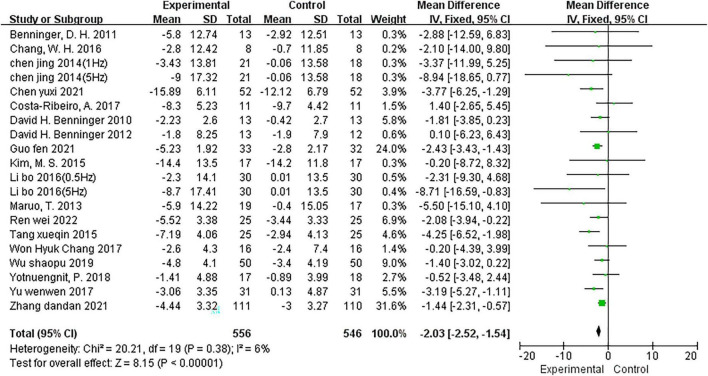
Meta-analyses of the effect of NIBS on UPDRS-III scores compared with the control group.

Two trials (Chen et al., 2014; Li, 2016) provided data for two comparisons (two intervention groups were included in the study design); therefore, 18 studies (Benninger et al., 2010, 2011, 2012; Maruo et al., 2013; Chen et al., 2014; Kim et al., 2015; Tang et al., 2015; Chang et al., 2016, 2017; Li, 2016; Costa-Ribeiro et al., 2017; Yu et al., 2017; Yotnuengnit et al., 2018; Wu et al., 2019; Chen and Li, 2021; Guo, 2021; Zhang and Mao, 2021; Ren and He, 2022) reported 20 comparisons with UPDRS-III scores. The fixed effects model was utilized to integrate the results (*I*^2^ = 6%). The meta-analysis showed a significant reduction in the UPDRS-III score for NIBS (MD = −2.03; 95% CI, −2.52 to −1.54; *P* < 0.00001; Figure 3) relative to the comparison group. Of the 20 comparisons, 15 (Benninger et al., 2011, 2012; Maruo et al., 2013; Chen et al., 2014; Kim et al., 2015; Tang et al., 2015; Chang et al., 2016; Li, 2016; Yu et al., 2017; Wu et al., 2019; Chen and Li, 2021; Guo, 2021; Ren and He, 2022) used the rTMS intervention, and five (Benninger et al., 2010; Chang et al., 2017; Costa-Ribeiro et al., 2017; Yotnuengnit et al., 2018; Zhang and Mao, 2021) adopted the tDCS intervention. Subgroup analysis based on this showed that both rTMS (MD = −2.54; 95% CI, −3.16 to −1.92; *P* < 0.00001; *I*^2^ = 0%) and tDCS (MD = −1.20; 95% CI, −1.99 to −0.40; *P* = 0.003; *I*^2^ = 0%) significantly reduced UPDRS-III scores.

#### Effects of NIBS on balance

**FIGURE 4:**
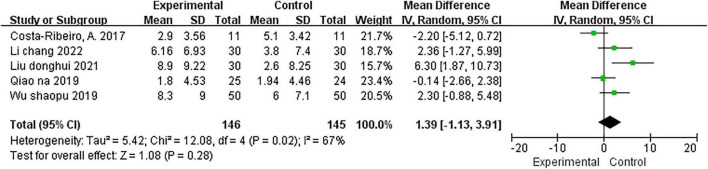
Meta-analyses of the effect of NIBS on balance compared with the control group.

The BBS was used to examine the effect of NIBS on balance in five studies (Costa-Ribeiro et al., 2017; Qiao et al., 2019; Wu et al., 2019; Liu and Zhang, 2021; Li et al., 2022); two (Wu et al., 2019; Li et al., 2022) of which used the rTMS intervention, and 3 (Costa-Ribeiro et al., 2017; Qiao et al., 2019; Liu and Zhang, 2021) used the tDCS intervention. Test for overall effect using a random effects model showed that the NIBS intervention had no significant effect on the balance (MD = 1.39; 95% CI, −1.13 to 3.91; *P* = 0.28; *I*^2^ = 67%; Figure 4) and subgroup analysis results for rTMS (MD = 2.33; 95% CI, −0.06 to 4.72; *P* = 0.06; *I*^2^ = 0%) and tDCS (MD = 0.97; 95% CI, −3.14 to 5.08; *P* = 0.64; *I*^2^ = 80%) also showed no significant effect.

#### Effects of NIBS on TUGT time

**FIGURE 5:**
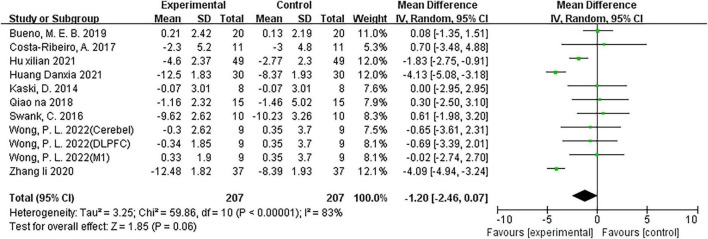
Meta-analyses of the effect of NIBS on TUGT time compared with the control group.

Nine studies (Kaski et al., 2014; Swank et al., 2016; Costa-Ribeiro et al., 2017; Qiao et al., 2018; Bueno et al., 2019; Zhang et al., 2020; Hu et al., 2021; Huang et al., 2021; Wong et al., 2022) including 11 comparisons reported the effect of NIBS on TUGT time, with two (Zhang et al., 2020; Huang et al., 2021) comparisons using rTMS interventions and nine (Kaski et al., 2014; Swank et al., 2016; Costa-Ribeiro et al., 2017; Qiao et al., 2018; Bueno et al., 2019; Hu et al., 2021; Wong et al., 2022) using tDCS interventions. The overall effect found that the NIBS intervention had no significant effect on TUGT time (MD = −1.20; 95% CI, −2.46 to 0.07; *P* = 0.06; *I*^2^ = 83%; Figure 5) compared with the control group. However, the subgroup analysis results for rTMS intervention (MD = −4.11; 95% CI, −4.74 to −3.47; *P* < 0.00001; *I*^2^ = 0%) and tDCS intervention (MD = −0.84; 95% CI, −1.48 to −0.21; *P* = 0.009; *I*^2^ = 12%)were showed a significant effect.

#### Effects of NIBS on walking parameters

**FIGURE 6:**
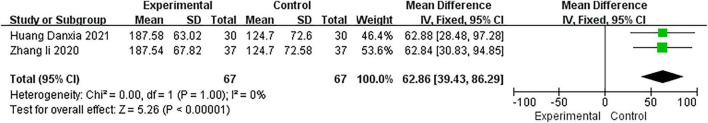
Meta-analyses of the effect of NIBS on 6MWD compared with the control group.

**FIGURE 7:**
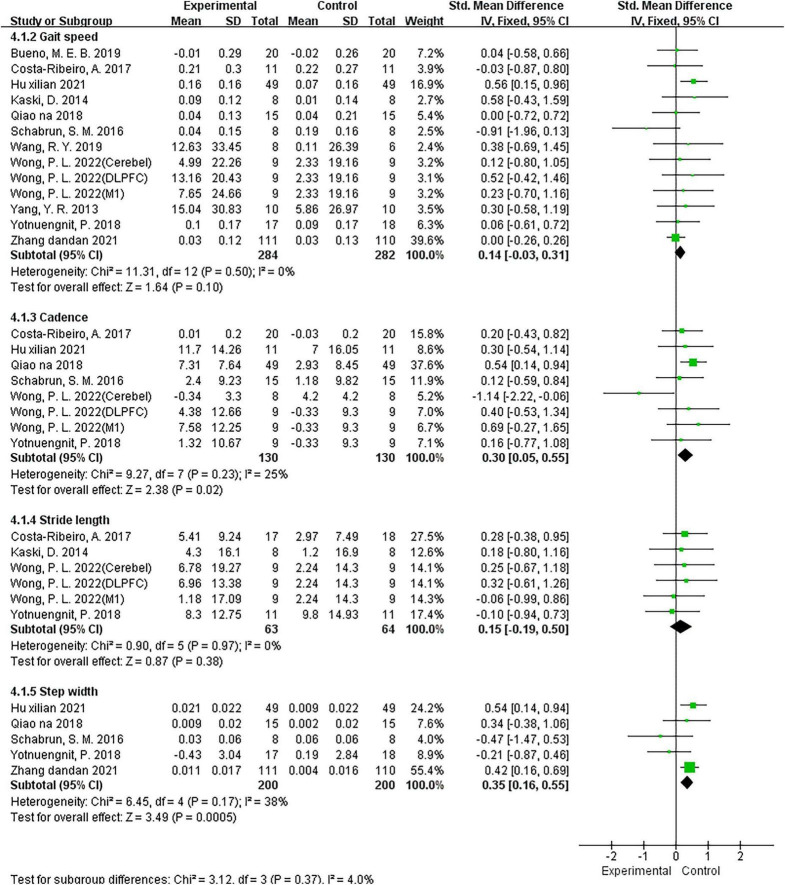
Meta-analyses of the effect of NIBS on other walking parameters compared with the control group.

To assess the effect of NIBS on walking ability in patients with PD, two studies (Zhang et al., 2020; Huang et al., 2021) used the 6MWD parameter, five studies (Schabrun et al., 2016; Qiao et al., 2018; Yotnuengnit et al., 2018; Hu et al., 2021; Zhang and Mao, 2021) used the step width parameter, four studies (Kaski et al., 2014; Costa-Ribeiro et al., 2017; Yotnuengnit et al., 2018; Wong et al., 2022) including six comparisons used the stride length parameter, 11 studies (Yang et al., 2013; Kaski et al., 2014; Schabrun et al., 2016; Costa-Ribeiro et al., 2017; Qiao et al., 2018; Yotnuengnit et al., 2018; Bueno et al., 2019; Wang et al., 2019; Hu et al., 2021; Zhang and Mao, 2021; Wong et al., 2022) including 13 comparisons used the gait speed parameter, and six studies (Schabrun et al., 2016; Costa-Ribeiro et al., 2017; Qiao et al., 2018; Yotnuengnit et al., 2018; Hu et al., 2021; Wong et al., 2022) including eight comparisons used the cadence parameter. The results of the analysis using a fixed model showed that compared with the control group, NIBS significantly improved 6MWT distance (MD = 62.86; 95% CI, 39.43–86.29; *P* < 0.00001; *I*^2^ = 0%; Figure 6), cadence (SMD = 0.3; 95% CI, 0.05 to 0.55; *P* = 0.02; *I*^2^ = 25%; Figure 7), and step width (SMD = 0.35; 95% CI, 0.16–0.55; *P* = 0.0005; *I*^2^ = 38%; Figure 7) but did not have a significant effect on gait speed (SMD = 0.14; 95% CI, −0.03 to 0.31; *P* = 0.10; *I*^2^ = 0%; Figure 7) and stride length (SMD = 0.15; 95% CI, −0.19 to 0.50; *P* = 0.38; *I*^2^ = 0%; Figure 7). On the other hand, the subgroup results for the gait speed also showed that neither the rTMS intervention (SMD = 0.34; 95% CI, −0.35 to 1.02; *P* = 0.34; *I*^2^ = 0%) nor the tDCS intervention (SMD = 0.13; 95% CI, −0.04 to 0.30; *P* = 0.15; *I*^2^ = 9%) had a significant effect on gait speed.

### Sensitivity analyses

Some results of this study had high heterogeneity, such as balance and TUGT time. One study (Liu and Zhang, 2021) was excluded from the sensitivity analysis for balance because its anode electrode placement was considerably different from the others. The pooled results of the remaining studies showed that the heterogeneity had become 47, but no statistical difference (MD = 0.28; 95% CI, −1.21 to 1.77; *P* = 0.71). Additionally, in the case of TUGT time, our subgroup findings have shown that the intervention type was the primary source of its heterogeneity.

### Publication bias

**FIGURE 8:**
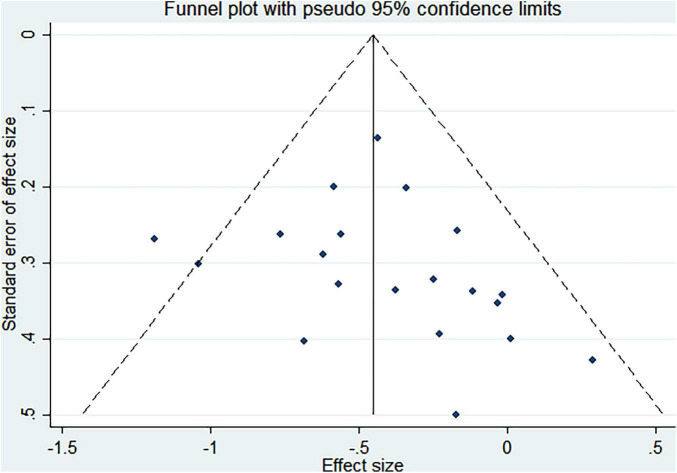
Funnel plot for UPDRS-III scores.

**FIGURE 9:**
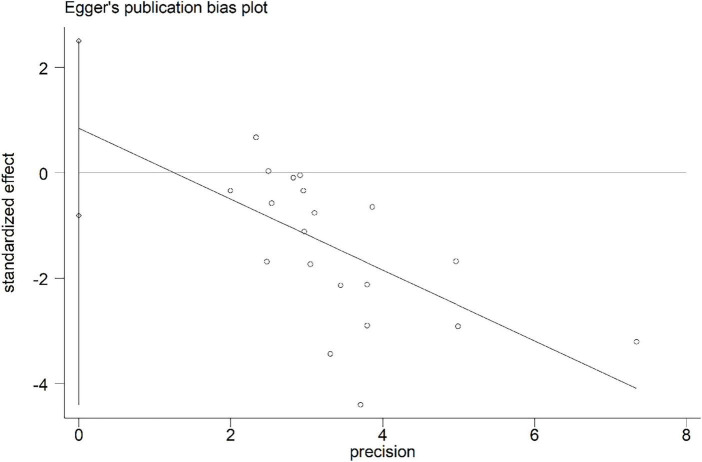
Egger’s publication bias plot for UPDRS-III scores.

**FIGURE 10:**
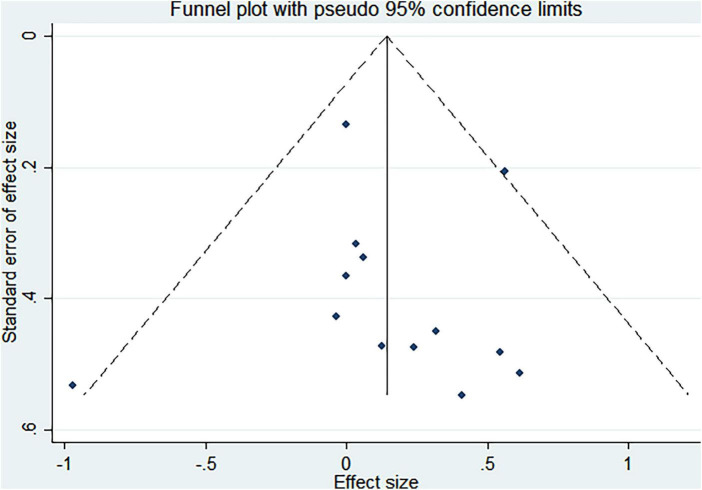
Funnel plot for gait speed.

**FIGURE 11:**
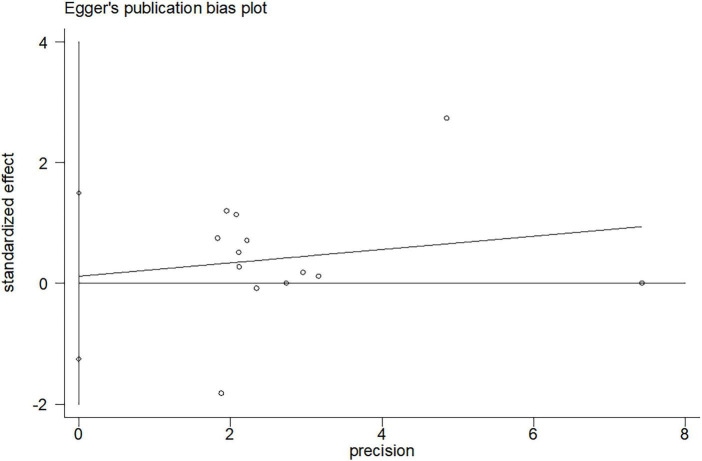
Egger’s publication bias plot for gait speed.

Funnel plots and Egger asymmetry tests were performed on outcome parameters (UPDRS-III scores and gait speed) containing more than ten studies. The results showed no publication bias (UPDRS-III scores: *p* = 0.298, Figures 8, 9; Gait speed: *p* = 0.853, Figures 10, 11).

### The results of *Z* tests

*Z*-tests referring to previous research methods (Zhang and Zhang, 2017; Higgins et al., 2019) were performed using R software to compare the overall effect of different subgroups. The results showed that the rTMS intervention was beneficial in reducing UPDRS-III scores and total TUGT time (*p* < 0.05) compared to the tDCS intervention (Table 4).

**TABLE 4:** Results of the comparison.

Outcome parameters	SMD/MD (95% CI)	*Z* value	*P*-value
UPDRS-III scores	−1.47 (−2.64, −0.299)	−2.46	0.0139
Gait speed	0.2 (−0.512, 0.912)	0.55	0.583
TUGT time	−1.09 (−2.17, −0.00917)	−1.98	0.0481
BBS scores	3.85 (−2.91, 10.6)	1.12	0.264

## Discussion

To our knowledge, this is the first meta-analysis to analyze the effectiveness of NIBS on the treatment of walking and balance ability in patients with PD and to compare the effect of rTMS and tDCS therapies. In this meta-analysis, we reviewed 32 studies, including 1,586 patients with PD. Our results found that NIBS improved UPDRS-III scores and walking ability variables such as step width, cadence, and 6MWT. In subgroup analyses across intervention types, UPDRS-III scores and TUGT significantly enhanced, and *Z* tests suggested that the rTMS intervention had more excellent effects than the tDCS intervention.

The UPDRS-III is a reliable and valid scale that correlates disease severity and quality of life with the assessment of motor symptoms in patients with PD (Schrag et al., 2000b; Movement Disorder Society Task Force on Rating Scales for Parkinson’s Disease, 2003). Our review thus included the UPDRS-III as one of the key outcome measures. Our findings showed that NIBS improved the UPDRS-III score by an average of 2.03 points. The magnitude of the effect for UPDRS-III scores found in our study is smaller than previous reviews (Chou et al., 2015; Zanjani et al., 2015; Chung and Mak, 2016), which reported improvements of 6.4, 3.8, and 2.7 points. This difference may be due to methodological differences. First, our study included rTMS and tDCS interventions, whereas the previous had only rTMS. Second, the previous study did not include studies published in Chinese. However, despite this, all reviews indicated a contribution of NIBS to UPDRS-III scores, demonstrating the credibility of our results.

On the other hand, our meta-analysis also found that NIBS improved walking ability variables such as step width, cadence, and 6MWT. Through the cortico-basal ganglia–thalamocortical circuit, NIBS may correct basal ganglia dysfunction, and this could be one way that NIBS helps patients with PD (Kim et al., 2019). Previous studies have shown (Shine et al., 2011; Kamble et al., 2014) that by directly increasing cortical excitability, NIBS increases the activity of the striatum and modulates inhibitory impulses of the globus pallidus internal, which leads to enhancing the patient’s motor performance. Another possible explanation could be that NIBS directly activates dopaminergic neurons in the striatum, providing endogenous dopamine (Strafella et al., 2003; Khedr et al., 2007). Regardless of the underlying cause, there appears to be little question that NIBS might enhance patients’ motor ability.

Interestingly, our results demonstrated that the effect of the NIBS on some of the patients’ outcomes (gait speed, TUGT time, balance, and stride length) in this study was not statistically significant. According to our analysis, two factors may have caused the above results. First, we consider that the diversity of the stimulation protocols utilized in each study was the cause of this result. Previous meta-analyses on rTMS and tDCS have confirmed the different effects of various stimulation protocols on study results (Lee et al., 2019; Nardone et al., 2020; de Oliveira et al., 2021; Nascimento et al., 2021; Pol et al., 2021; Deng et al., 2022; Krogh et al., 2022). Although our results did not reveal a statistically significant relationship between NIBS and gait speed, balance, or stride length, this does not mean that NIBS does not affect these outcomes. The various stimulation protocols used in the study should be considered in the future when interpreting the results. Further investigation is needed to assess how NIBS’s positive effects can be achieved and determine the best stimulation protocol, including stimulation intensity, duration, and area of stimulation, among others. Second, we believe that the quality of the included studies was another reason for this result. Some studies had a high risk of selection bias, detection bias, and other biases (Figure 2), which may have influenced our results. In light of this, future studies should be further investigated.

In our study, further subgroup analyses were performed for outcomes with large heterogeneity in the pooled results (e.g., gait speed, BBS scores, UPDRS-III scores, and TUGT). Both rTMS and tDCS were shown to have significant elevation effects on UPDRS-III scores and TUGT but no influence on gait speed or BBS scores. A previous meta-analysis of five randomized clinical trials showed that increasing tDCS did not provide additional benefits for gait speed and stride length, which is consistent with our results. The previous (Nascimento et al., 2021) explanation was that NIBS was a superficial stimulation whose influence on walking ability has not yet been reached. However, unlike previous studies, our study showed that NIBS had a significant effect on cadence in patients with PD in our review. Our results lead us to believe that superficial stimulation is not the main reason for the differences in the results of our review. Future studies are needed to investigate the causes of the variations in study outcomes due to other factors, such as disease type and intervention protocols. On the other hand, we only included five studies that measured balance by BBS scores, including two rTMS studies and three tDCS studies. The smaller number of literature may have caused a larger bias in the outcome, so more research is needed to determine how NIBS affects balance ability.

It is worth noting that our results showed that rTMS was more significant in improving UPDRS-III scores and TUGT time compared to tDCS. As the two most prevalent NIBS techniques, rTMS and tDCS have been extensively used in the treatment of motor abilities of patients, but their fundamental principles are different, and each has its advantages. It is commonly believed that anodal tDCS enhances the function of the underlying areas of the cortex, whereas cathodal tDCS has a suppressive impact (Nitsche and Paulus, 2011). Depending on the stimulation frequency, which ranges from 1 to 50 Hz, TMS may either cause an increase or reduction in cortical excitability (Koch, 2013). The home application is possible in the case of tDCS (Begemann et al., 2020). So, we recommended that future therapy choices be based on the patient’s actual state. In addition, due to the few article numbers included in the outcome parameters, there may be a substantial bias in the review findings, and future large-scale clinical randomized trials should be conducted to confirm the intervention benefits of NIBS.

Although we have a comprehensive analysis and assessed all eligible studies, it still has some limitations. First, the diversity of the protocols used in each study may have led to variability in the results. Although our results demonstrate the effect of NIBS on patients with PD, the few studies and the heterogeneity of stimulation protocols should be considered when interpreting the results. Second, most of the studies showed only randomized trials but no specific methods of random sequence generation, RCTs of allocation concealment, or blinding of outcome assessment. Many of the included RCTs were generally of low methodological quality and may have a high risk of bias. Finally, some of the studies included in the meta-analysis were published by the same research center, possibly overlapping data.

Further investigation is needed to assess how the positive effects of NIBS can be maintained over time and to determine the optimal stimulation regimen, including stimulation intensity, duration, and sites. Moreover, it is also necessary to examine the effects of NIBS on walking ability and balance according to disease type and intervention type. NIBS’ value for walking and balance ability in PD deserves special attention in future studies.

## Conclusion

In summary, the current systematic review and meta-analysis provided evidence that NIBS intervention has several benefits for walking in patients with PD, but further research is needed to improve balance ability. Moreover, rTMS significantly improved UPDRS-III scores and TUGT time compared to tDCS. These findings provide important clinical implications to researchers and clinicians in the utility of NIBS as a potential treatment protocol. Future studies should look at the best target brain regions, stimulation intensity, timing, and type of intervention to better understand the effects of NIBS treatment on walking and balance ability. They should also look at the best protocol for rehabilitation for patients with PD’s balance and walking ability.

## Data availability statement

The original contributions presented in this study are included in the article/supplementary material, further inquiries can be directed to the corresponding author.

## Author contributions

XZ did the study conception, design, data collection, statistics, and writing. XZ, FJ, YL, WL, and QL did the data collection and participated in writing. JT, PG, HT, JZ, and XH participated in the study conception, design, statistics, and writing. All authors read and approved the final version of the manuscript.

## References

[B1] Bayle N. Patel A. S. Crisan D. Guo L. J. Hutin E. Weisz D. J. (2016). Contribution of step length to increase walking and turning speed as a marker of Parkinson’s disease progression. *PLoS One* 11:e0152469. 10.1371/journal.pone.0152469 27111531 PMC4844147

[B2] Begemann M. J. Brand B. A. Ćurčić-Blake B. Aleman A. Sommer I. E. (2020). Efficacy of non-invasive brain stimulation on cognitive functioning in brain disorders: A meta-analysis. *Psychol. Med.* 50 2465–2486. 10.1017/S0033291720003670 33070785 PMC7737055

[B3] Benninger D. H. Hallett M. (2015). Non-invasive brain stimulation for Parkinson’s disease: Current concepts and outlook 2015. *NeuroRehabilitation* 37 11–24. 10.3233/NRE-151237 26409690

[B4] Benninger D. H. Berman B. D. Houdayer E. Pal N. Luckenbaugh D. A. Schneider L. (2011). Intermittent theta-burst transcranial magnetic stimulation for treatment of Parkinson disease. *Neurology* 76 601–609. 10.1212/WNL.0b013e31820ce6bb 21321333 PMC3053339

[B5] Benninger D. H. Iseki K. Kranick S. Luckenbaugh D. A. Houdayer E. Hallett M. (2012). Controlled study of 50-Hz repetitive transcranial magnetic stimulation for the treatment of Parkinson disease. *Neurorehabil. Neural Repair* 26 1096-1105. 10.1177/1545968312445636 22593114 PMC4169897

[B6] Benninger D. H. Lomarev M. Lopez G. Wassermann E. M. Li X. Considine E. (2010). Transcranial direct current stimulation for the treatment of Parkinson’s disease. *J. Neurol. Neurosurg. Psychiatry* 81 1105–111. 10.1136/jnnp.2009.202556 20870863 PMC4162743

[B7] Bueno M. E. B. do Nascimento Neto L. I. Terra M. B. Barboza N. M. Okano A. H. Smaili S. M. (2019). Effectiveness of acute transcranial direct current stimulation on non-motor and motor symptoms in Parkinson’s disease. *Neurosci. Lett.* 696 46–51. 10.1016/j.neulet.2018.12.017 30553865

[B8] Chang W. H. Kim M. S. Cho J. W. Youn J. Kim Y. K. Kim S. W. (2016). Effect of cumulative repetitive transcranial magnetic stimulation on freezing of gait in patients with atypical Parkinsonism: A pilot study. *J. Rehabil. Med.* 48 824–828. 10.2340/16501977-2140 27670977

[B9] Chang W. H. Kim M. S. Park E. Cho J. W. Youn J. Kim Y. K. (2017). Effect of dual-mode and dual-site noninvasive brain stimulation on freezing of gait in patients with Parkinson disease. *Arch. Phys. Med. Rehabil.* 98 1283–1290. 10.1016/j.apmr.2017.01.011 28193533

[B10] Chen J. Zhang C. G. Zhang H. B. Zhang Q. C. Zhou S. H. Yang J. X. (2014). Clinical observation of high-frequency and low-frequency repetitive transcranial magnetic stimulation in the treatment of Parkinson’s disease. *Chin. J. Rehabil. Med.* 29 464–467.

[B11] Chen Y. X. Li M. L. (2021). Effect of low-frequency repetitive transcranial magnetic stimulation combined with dobutamine in patients with autonomic dysfunction in Parkinson’s disease. *Med. J. Chin. Peoples Health* 33 31–33.

[B12] Chou Y. H. Hickey P. T. Sundman M. Song A. W. Chen N. K. (2015). Effects of repetitive transcranial magnetic stimulation on motor symptoms in Parkinson disease: A systematic review and meta-analysis. *JAMA Neurol.* 72 432–440. 10.1001/jamaneurol.2014.4380 25686212 PMC4425190

[B13] Chung C. L. Mak M. K. (2016). Effect of repetitive transcranial magnetic stimulation on physical function and motor signs in Parkinson’s disease: A systematic review and meta-analysis. *Brain Stimul.* 9 475–487. 10.1016/j.brs.2016.03.017 27117282

[B14] Chung K. C. Burns P. B. Kim H. M. (2006). A practical guide to meta-analysis. *J. Hand Surg.* 31 1671–1678. 10.1016/j.jhsa.2006.09.002 17145390

[B15] Connolly B. S. Lang A. E. (2014). Pharmacological treatment of Parkinson disease: A review. *JAMA* 311 1670–1683. 10.1001/jama.2014.3654 24756517

[B16] Costa-Ribeiro A. Maux A. Bosford T. Aoki Y. Castro R. Baltar A. (2017). Transcranial direct current stimulation associated with gait training in Parkinson’s disease: A pilot randomized clinical trial. *Dev. Neurorehabil.* 20 121–128. 10.3109/17518423.2015.1131755 26864140

[B17] de Oliveira P. C. A. de Araújo T. A. B. Machado D. Rodrigues A. C. Bikson M. Andrade S. M. (2021). Transcranial direct current stimulation on Parkinson’s disease: Systematic review and meta-analysis. *Front. Neurol.* 12:794784. 10.3389/fneur.2021.794784 35082749 PMC8785799

[B18] Deng S. Dong Z. Pan L. Liu Y. Ye Z. Qin L. (2022). Effects of repetitive transcranial magnetic stimulation on gait disorders and cognitive dysfunction in Parkinson’s disease: A systematic review with meta-analysis. *Brain Behav.* 12:e2697. 10.1002/brb3.2697 35862217 PMC9392523

[B19] Elsner B. Kugler J. Pohl M. Mehrholz J. (2016). Transcranial direct current stimulation (tDCS) for idiopathic Parkinson’s disease. *Cochrane Database Syst. Rev.* 7:Cd010916. 10.1002/14651858.CD010916.pub2 27425786 PMC6457946

[B20] Faggiani E. Benazzouz A. (2017). Deep brain stimulation of the subthalamic nucleus in Parkinson’s disease: From history to the interaction with the monoaminergic systems. *Prog. Neurobiol.* 151 139–156. 10.1016/j.pneurobio.2016.07.003 27412110

[B21] Feingold A. (2009). Effect sizes for growth-modeling analysis for controlled clinical trials in the same metric as for classical analysis. *Psychol. Methods* 14 43–53. 10.1037/a0014699 19271847 PMC2712654

[B22] Gao P. Tang F. Liu W. He K. Mo Y. (2021). Effect of liuzijue qigong on patients with stable chronic obstructive pulmonary disease: A systematic review and meta-analysis. *Medicine (Baltimore)* 100:e27344. 10.1097/MD.0000000000027344 34731105 PMC8519198

[B23] GBD 2016 Parkinson’s Disease Collaborators (2018). Global, regional, and national burden of Parkinson’s disease, 1990-2016: A systematic analysis for the global burden of disease study 2016. *Lancet Neurol.* 17 939–953. 10.1016/S1474-4422(18)30295-3 30287051 PMC6191528

[B24] Guo F. (2021). Efficacy of low-frequency repetitive transcranial magnetic stimulation combined with pramipexole in the treatment of early Parkinson’s patients. *Heilongjiang Med. Pharm.* 44 39–40.

[B25] Helmich R. C. Siebner H. R. Bakker M. Münchau A. Bloem B. R. (2006). Repetitive transcranial magnetic stimulation to improve mood and motor function in Parkinson’s disease. *J. Neurol. Sci.* 248 84–96. 10.1016/j.jns.2006.05.009 16793065

[B26] Higgins J. P. Altman D. G. Gøtzsche P. C. Jüni P. Moher D. Oxman A. D. (2011). The Cochrane collaboration’s tool for assessing risk of bias in randomised trials. *BMJ* 343:d5928. 10.1136/bmj.d5928 22008217 PMC3196245

[B27] Higgins J. P. Thomas J. Chandler J. Cumpston M. Li T. Page M. J. (2019). *Cochrane handbook for systematic reviews of interventions.* Hoboken, NJ: John Wiley & Sons. 10.1002/9781119536604

[B28] Hu X. L. Xue C. P. Liu Z. S. (2021). The effect of transcranial direct current stimulation-assisted functional rehabilitation training on the rehabilitation of patients with Parkinson’s disease Chinese. *J. Gerontol.* 41 3724–3727.

[B29] Huang D. H. Wu C. S. Zhuang Y. H. (2021). Analysis of the application effect of repetitive transcranial magnetic stimulation + rehabilitation training in patients with Parkinson’s disease and the impact of cognitive function score. *China For. Med. Treat.* 40 39–42.

[B30] Kamble N. Netravathi M. Pal P. K. (2014). Therapeutic applications of repetitive transcranial magnetic stimulation (rTMS) in movement disorders: A review. *Parkinsonism Relat. Disord.* 20 695–707. 10.1016/j.parkreldis.2014.03.018 24726453

[B31] Kaski D. Dominguez R. O. Allum J. H. Islam A. F. Bronstein A. M. (2014). Combining physical training with transcranial direct current stimulation to improve gait in Parkinson’s disease: A pilot randomized controlled study. *Clin. Rehabil.* 28 1115–1124. 10.1177/0269215514534277 24849794

[B32] Khedr E. M. Rothwell J. C. Shawky O. A. Ahmed M. A. Foly N. Hamdy A. (2007). Dopamine levels after repetitive transcranial magnetic stimulation of motor cortex in patients with Parkinson’s disease: Preliminary results. *Mov. Disord.* 22 1046–1050. 10.1002/mds.21460 17575584

[B33] Kim M. S. Chang W. H. Cho J. W. Youn J. Kim Y. K. Kim S. W. (2015). Efficacy of cumulative high-frequency rTMS on freezing of gait in Parkinson’s disease. *Restor. Neurol. Neurosci.* 33 521–530. 10.3233/RNN-140489 26409410 PMC4923757

[B34] Kim Y. W. Shin I. S. Moon H. I. Lee S. C. Yoon S. Y. (2019). Effects of non-invasive brain stimulation on freezing of gait in Parkinsonism: A systematic review with meta-analysis. *Parkinsonism Relat. Disord.* 64 82–89. 10.1016/j.parkreldis.2019.02.029 30902526

[B35] Koch G. (2013). Do studies on cortical plasticity provide a rationale for using non-invasive brain stimulation as a treatment for Parkinson’s disease patients? *Front. Neurol.* 4:180. 10.3389/fneur.2013.00180 24223573 PMC3818583

[B36] Krogh S. Jønsson A. B. Aagaard P. Kasch H. (2022). Efficacy of repetitive transcranial magnetic stimulation for improving lower limb function in individuals with neurological disorders: A systematic review and meta-analysis of randomized sham-controlled trials. *J. Rehabil. Med.* 54:jrm00256. 10.2340/jrm.v53.1097 34913062 PMC8862648

[B37] LaHue S. C. Comella C. L. Tanner C. M. (2016). The best medicine? The influence of physical activity and inactivity on Parkinson’s disease. *Mov. Disord.* 31 1444–1454. 10.1002/mds.26728 27477046 PMC9491025

[B38] Lee H. K. Ahn S. J. Shin Y. M. Kang N. Cauraugh J. H. (2019). Does transcranial direct current stimulation improve functional locomotion in people with Parkinson’s disease? A systematic review and meta-analysis. *J. Neuroeng. Rehabil.* 16:84. 10.1186/s12984-019-0562-4 31286974 PMC6615099

[B39] Li B. (2016). Evaluation on curative effect and safety of low frequency and high frequency repetitive transcranial magnetic stimulation in treatment of Parkinson disease. *China For. Med. Treat.* 35 188–190.

[B40] Li C. Wu J. Zhou M. Pang G. F. (2022). Evaluation of the efficacy of repetitive transcranial magnetic stimulation combined with Praxeol in the treatment of senile Parkinson’s disease. *Chin. J. Geriatr. Care* 20 57–60.

[B41] Liu D. H. Zhang D. H. (2021). Efficacy of transcranial direct current stimulation to improve balance disorders in patients with Parkinson’s disease. *Chin. J. Med.* 56 765–768.

[B42] Maruo T. Hosomi K. Shimokawa T. Kishima H. Oshino S. Morris S. (2013). High-frequency repetitive transcranial magnetic stimulation over the primary foot motor area in Parkinson’s disease. *Brain Stimul.* 6 884–891. 10.1016/j.brs.2013.05.002 23769414

[B43] Mehrholz J. Kugler J. Storch A. Pohl M. Elsner B. Hirsch K. (2015). Treadmill training for patients with Parkinson’s disease. *Cochrane Database Syst. Rev.* 22:Cd007830. 10.1002/14651858.CD007830.pub3 26297797

[B44] Moher D. Liberati A. Tetzlaff J. Altman D. G. (2009). Preferred reporting items for systematic reviews and meta-analyses: The PRISMA statement. *J. Clin. Epidemiol.* 62 1006–1012. 10.1016/j.jclinepi.2009.06.005 19631508

[B45] Morgan J. C. Currie L. J. Harrison M. B. Bennett J. P. Jr. Trugman J. M. Wooten G. F. (2014). Mortality in levodopa-treated Parkinson’s disease. *Parkinsons Dis.* 2014:426976. 10.1155/2014/426976 24616821 PMC3927757

[B46] Morgante L. Morgante F. Moro E. Epifanio A. Girlanda P. Ragonese P. (2007). How many Parkinsonian patients are suitable candidates for deep brain stimulation of subthalamic nucleus? Results of a questionnaire. *Parkinsonism Relat. Disord.* 13 528–531. 10.1016/j.parkreldis.2006.12.013 17347021

[B47] Movement Disorder Society Task Force on Rating Scales for Parkinson’s Disease (2003). The unified Parkinson’s disease rating scale (UPDRS): Status and recommendations. *Mov. Disord.* 18 738–750. 10.1002/mds.10473 12815652

[B48] Nardone R. Versace V. Brigo F. Golaszewski S. Carnicelli L. Saltuari L. (2020). Transcranial magnetic stimulation and gait disturbances in Parkinson’s disease: A systematic review. *Neurophysiol. Clin.* 50 213–225. 10.1016/j.neucli.2020.05.002 32620273

[B49] Nascimento L. R. do Carmo W. A. de Oliveira G. P. Areas F. Dias F. M. V. (2021). Transcranial direct current stimulation provides no clinically important benefits over walking training for improving walking in Parkinson’s disease: A systematic review. *J. Physiother.* 67 190–196. 10.1016/j.jphys.2021.06.003 34147400

[B50] Nijkrake M. J. Keus S. H. Kalf J. G. Sturkenboom I. H. Munneke M. Kappelle A. C. (2007). Allied health care interventions and complementary therapies in Parkinson’s disease. *Parkinsonism Relat. Disord.* 13 (Suppl. 3) S488–S494. 10.1016/S1353-8020(08)70054-3 18267288

[B51] Nitsche M. A. Paulus W. (2011). Transcranial direct current stimulation–update 2011. *Restor. Neurol. Neurosci.* 29 463–492. 10.3233/RNN-2011-0618 22085959

[B52] Obeso J. A. Stamelou M. Goetz C. G. Poewe W. Lang A. E. Weintraub D. (2017). Past, present, and future of Parkinson’s disease: A special essay on the 200th anniversary of the shaking palsy. *Mov. Disord.* 32 1264–1310. 10.1002/mds.27115 28887905 PMC5685546

[B53] Pol F. Salehinejad M. A. Baharlouei H. Nitsche M. A. (2021). The effects of transcranial direct current stimulation on gait in patients with Parkinson’s disease: A systematic review. *Transl. Neurodegener.* 10:22. 10.1186/s40035-021-00245-2 34183062 PMC8240267

[B54] Qiao N. Lu J. J. Guo Y. L. Zheng W. H. Yan T. B. (2019). Effects of transcranial direct current stimulation on balance function in patients with Parkinson’s disease. *Rehabil. Med.* 29 22–26.

[B55] Qiao N. Yan T. B. Lu J. J. Guo Y. L. Zheng W. H. (2018). Effect of transcranial direct current stimulation on the walking function of patients with early Parkinson’s disease: A randomized and controlled trial. *Chin. J. Phys. Med. Rehabil.* 40 509–512.

[B56] Rascol O. Payoux P. Ferreira J. Brefel-Courbon C. (2002). The management of patients with early Parkinson’s disease. *Parkinsonism Relat. Disord.* 9 61–67. 10.1016/S1353-8020(02)00045-7 12217623

[B57] Ren W. He Y. Q. (2022). Efficacy of repetitive transcranial magnetic stimulation combined with rehabilitation training in patients with Parkinson’s disease. *Ningxia Med. J.* 44 147–149.

[B58] Schabrun S. M. Lamont R. M. Brauer S. G. (2016). Transcranial direct current stimulation to enhance dual-task gait training in Parkinson’s disease: A pilot RCT. *PLoS One* 11:e0158497. 10.1371/journal.pone.0158497 27359338 PMC4928827

[B59] Schlenstedt C. Shalash A. Muthuraman M. Falk D. Witt K. Deuschl G. (2017). Effect of high-frequency subthalamic neurostimulation on gait and freezing of gait in Parkinson’s disease: A systematic review and meta-analysis. *Eur. J. Neurol.* 24 18–26. 10.1111/ene.13167 27766724

[B60] Schrag A. Jahanshahi M. Quinn N. (2000a). How does Parkinson’s disease affect quality of life? A comparison with quality of life in the general population. *Mov. Disord.* 15 1112–1118. 10.1002/1531-8257(200011)15:6<1112::AID-MDS1008>3.0.CO;2-A 11104193

[B61] Schrag A. Jahanshahi M. Quinn N. (2000b). What contributes to quality of life in patients with Parkinson’s disease? *J. Neurol. Neurosurg. Psychiatry* 69 308–312. 10.1136/jnnp.69.3.308 10945804 PMC1737100

[B62] Shine J. M. Naismith S. L. Lewis S. J. (2011). The pathophysiological mechanisms underlying freezing of gait in Parkinson’s disease. *J. Clin. Neurosci.* 18 1154–1157. 10.1016/j.jocn.2011.02.007 21724402

[B63] Sterne J. A. Sutton A. J. Ioannidis J. P. Terrin N. Jones D. R. Lau J. (2011). Recommendations for examining and interpreting funnel plot asymmetry in meta-analyses of randomised controlled trials. *BMJ* 343:d4002. 10.1136/bmj.d4002 21784880

[B64] Strafella A. P. Paus T. Fraraccio M. Dagher A. (2003). Striatal dopamine release induced by repetitive transcranial magnetic stimulation of the human motor cortex. *Brain* 126(Pt 12) 2609–2615. 10.1093/brain/awg268 12937078

[B65] Swank C. Mehta J. Criminger C. (2016). Transcranial direct current stimulation lessens dual task cost in people with Parkinson’s disease. *Neurosci. Lett.* 626 1–5. 10.1016/j.neulet.2016.05.010 27181509

[B66] Tang X. Q. Deng J. G. Song T. Liu J. Zhang C. J. Wu Y. C. (2015). Effect of low frequency repetitive transcranial magnetic stimulation on dysfunction and depressive symptoms of Parkinson disease patients. *China Modern Doct.* 53 92–94.

[B67] Tomlinson C. L. Patel S. Meek C. Herd C. P. Clarke C. E. Stowe R. (2013). Physiotherapy versus placebo or no intervention in Parkinson’s disease. *Cochrane Database Syst. Rev.* 2013:Cd002817. 10.1002/14651858.CD002817.pub4 22895932

[B68] Wang R. Y. Wang F. Y. Huang S. F. Yang Y. R. (2019). High-frequency repetitive transcranial magnetic stimulation enhanced treadmill training effects on gait performance in individuals with chronic stroke: A double-blinded randomized controlled pilot trial. *Gait Posture* 68 382–387. 10.1016/j.gaitpost.2018.12.023 30586670

[B69] Wong P. L. Yang Y. R. Huang S. F. Fuh J. L. Chiang H. L. Wang R. Y. (2022). Transcranial direct current stimulation on different targets to modulate cortical activity and dual-task walking in individuals with Parkinson’s disease: A double blinded randomized controlled trial. *Front. Aging Neurosci.* 14:807151. 10.3389/fnagi.2022.807151 35197844 PMC8859467

[B70] Wu S. P. Li X. Qi Y. W. Wang H. Zhang W. S. Yang H. Q. (2019). Effects of transcranial magnetic stimulation on the motor and non-motor symptoms in Parkinson’s disease. *Chin. J. Phys. Med. Rehabil.* 41 338–343.

[B71] Yang Y. R. Tseng C. Y. Chiou S. Y. Liao K. K. Cheng S. J. Lai K. L. (2013). Combination of rTMS and treadmill training modulates corticomotor inhibition and improves walking in Parkinson disease: A randomized trial. *Neurorehabil. Neural Repair* 27 79–86. 10.1177/1545968312451915 22785003

[B72] Yotnuengnit P. Bhidayasiri R. Donkhan R. Chaluaysrimuang J. Piravej K. (2018). Effects of transcranial direct current stimulation plus physical therapy on gait in patients with Parkinson disease: A randomized controlled trial. *Am. J. Phys. Med. Rehabil.* 97 7–15. 10.1097/PHM.0000000000000783 28650857

[B73] Yu W. W. Li Z. G. Sun H. R. Lu A. X. Zhang J. S. (2017). Effects of high frequency repetitive transcranial magnetic stimulation on motor fuction, cognitivefunction and autonomic disorder in patients with early Parkinson’s disease. *Acta Acad. Med. Weifang* 39 288–290.

[B74] Zanjani A. Zakzanis K. K. Daskalakis Z. J. Chen R. (2015). Repetitive transcranial magnetic stimulation of the primary motor cortex in the treatment of motor signs in Parkinson’s disease: A quantitative review of the literature. *Mov. Disord.* 30 750–758. 10.1002/mds.26206 25786995

[B75] Zhang D. D. Mao J. P. (2021). Effects of repeated transcranial direct current stimulation on trunk electromyography, walking function and quality of life in PD patients. *Chin. J. Health Care Med.* 23 465–468.

[B76] Zhang L. Li F. H. Xu P. F. (2020). Effects of rTMS combined with rehabilitation training on patients with Parkinson disease. *Med. J. Chin. Peoples Health* 32 57–59.

[B77] Zhang T. S. Zhang S. X. (2017). How to compare summary estimates of different subgroups in meta-analysis. *Chin. J. Evid. Based Med.* 17 1465–1470.

